# Metabolomics and Ionomics of Potato Tuber Reveals an Influence of Cultivar and Market Class on Human Nutrients and Bioactive Compounds

**DOI:** 10.3389/fnut.2018.00036

**Published:** 2018-05-23

**Authors:** Jacqueline M. Chaparro, David G. Holm, Corey D. Broeckling, Jessica E. Prenni, Adam L. Heuberger

**Affiliations:** ^1^Department of Horticulture and Landscape Architecture, Colorado State University, Fort Collins, CO, United States; ^2^Proteomics and Metabolomics Facility, Colorado State University, Fort Collins, CO, United States; ^3^Department of Soil and Crop Sciences, Colorado State University, Fort Collins, CO, United States

**Keywords:** *Solanum tuberosum* L., potato, bioactive compounds, nutrients, ionomics, non-targeted metabolomics, human health

## Abstract

Potato (*Solanum tuberosum* L.) is an important global food crop that contains phytochemicals with demonstrated effects on human health. Understanding sources of chemical variation of potato tuber can inform breeding for improved health attributes of the cooked food. Here, a comprehensive metabolomics (UPLC- and GC-MS) and ionomics (ICP-MS) analysis of raw and cooked potato tuber was performed on 60 unique potato genotypes that span 5 market classes including russet, red, yellow, chip, and specialty potatoes. The analyses detected 2,656 compounds that included known bioactives (43 compounds), nutrients (42), lipids (76), and 23 metals. Most nutrients and bioactives were partially degraded during cooking (44 out of 85; 52%), however genotypes with high quantities of bioactives remained highest in the cooked tuber. Chemical variation was influenced by genotype and market class. Specifically, ~53% of all detected compounds from cooked potato varied among market class and 40% varied by genotype. The most notable metabolite profiles were observed in yellow-flesh potato which had higher levels of carotenoids and specialty potatoes which had the higher levels of chlorogenic acid as compared to the other market classes. Variation in several molecules with known association to health was observed among market classes and included vitamins (e.g., pyridoxal, ~2-fold variation), bioactives (e.g., chlorogenic acid, ~40-fold variation), medicinals (e.g., kukoamines, ~6-fold variation), and minerals (e.g., calcium, iron, molybdenum, ~2-fold variation). Furthermore, more metabolite variation was observed within market class than among market class (e.g., α-tocopherol, ~1-fold variation among market class vs. ~3-fold variation within market class). Taken together, the analysis characterized significant metabolite and mineral variation in raw and cooked potato tuber, and support the potential to breed new cultivars for improved health traits.

## Introduction

Chronic diseases such as heart disease, diabetes, cancer, and obesity are a global problem, accounting for 2/3 of global mortality, and rates of these conditions have been shown to be twice as high in low/middle income countries (1). The impact of chronic diseases is strongly influenced by lifestyle choices such as exercise and diet (1). While diet may not be a sole or even major component of disease prevention or interception, there is a clear role of nutrients and other bioactive compounds in the initiation, development, and severity of chronic diseases (2–5). This is supported by epidemiological studies that highlight the importance of food, diet, and nutrition to prevent and control development of such diseases (5–7). Plant foods provide two types of compounds with effects on health: nutrients (required for human metabolism and development, such as vitamins and minerals) and bioactive compounds (herein referred to as “bioactives”) that have physiological, behavioral, and/or immunological effects but are not known to be essential to sustain life (8–10).

Potato (*Solanum tuberosum* L.) is an important source of nutrients and bioactives for the global population. It is the third most consumed food crop and diet staple for over one billion people (11). Potatoes are grown in nearly all nations, and production in developing nations equaled the developed world in 2005 (11, 12). The potato tuber contains a diverse set of nutrients and bioactive compounds with clear effects on preventing and combating chronic diseases such as hypertension, cancer, diabetes, and heart disease (13–23). The tuber is a nutrient-dense food, which means that it provides a greater percentage of nutrients than its estimated 100 calories per serving (12, 24, 25). For example, 100 g of baked potato (97 calories) contains 15% of the recommended amounts of vitamin B6, 16% of potassium, 9% of magnesium, 6% of iron, and 4% of pantothenic acid (12, 26). Potato tuber also contains several bioactives including polyphenolics (e.g., chlorogenic acid, methylbelliferones, and the flavonoids apigenin, rutin, and kaempferol 3-O-rutinoside), terpenes (e.g., the carotenoids lutein and neoxanthin), polyamines (e.g., kukoamines), and alkaloids (e.g., calystegines, solanine, tomatine, and chaconine), which have demonstrated activity against cancer (27–32), heart disease (31, 33, 34), hypertension (33, 35–37), diabetes (38–42), Parkinson's disease (43), Alzheimer's disease (44, 45), and obesity (46, 47). Glycoalkaloids are most commonly found in the *Solanaceae* family which includes potatoes (48). While toxic in large doses, glycoalkaloids can reduce serum cholesterol and have anticarcinogenic effects (49–52), for example by disrupting cell membranes and modulating calcium ion concentrations (53).

As a plant and food, potato has a vast biodiversity with over 5,000 documented cultivars and more than 100 wild potato species (54). In U.S. potato breeding for commercial production, this biodiversity has enabled the formation of distinct market classes with unique tuber phenotypes. These market classes include: *russets* (oblong-long shape for baking and frying, used in the fresh and processing markets), *reds* (oval-round shape for fresh market, higher sugar content), *yellows* (high carotenoids for yellow internal flesh, fresh market), *chips* (quality, density and round shape for chip processing), and *specialties* (non-traditional shapes, mixed colors, heirlooms). Variation within each market class consists of unique cultivars, which are potato genotypes released from breeding programs for commercial use. Additional genetic diversity can be found in potato breeding programs within advanced lines, which are potato genotypes that have undergone selection for critical quality traits, but have not yet been released to the market.

The large genotypic and phenotypic diversity in potato supports the hypothesis that tubers will vary widely in their content of nutrients and bioactives. Previous studies have demonstrated genetic control over nutrients and bioactives in foods such as rice, tomato, and potato (55–59). Studies on diverse potato populations have revealed variation in carotenoids, total phenolics, iron and zinc, chlorogenic acid, caffeic acid, rutin, and kaempferol (59, 60). Similar results were obtained in a study that analyzed 25 potato genotypes from the Texas Potato Variety Development Program and found that potato genotype significantly influenced phenolic and carotenoid content (61).

Current research highlights the utility of metabolomics and ionomics as high-throughput methods to profile variation in nutrients and bioactive compounds in foods. In these studies, mass spectrometry is used to profile small molecules (i.e., metabolites < 1,200 Da) and elements (minerals) (62–64). For example, metabolomics and ionomics have been utilized to evaluate diversity in the nutrient content and health traits of plant-based foods for up to 21 minerals and thousands of metabolites in rice, tomato, wheat, ají, cucumber, eggplant, beans, and others (55, 56, 63–69). Several studies have shown an influence of plant genotype on metabolite content of foods, including wheat (phenolics and sterols) (69) and rice (phenolics, tocopherols, phytosterols, fatty acids) (55). Other studies have evaluated how plant genetics lead to differences in mineral content of vegetable crops (essential minerals) (68), rice (Ca, Cu, K, Na, Zn) (70), maize (71, 72), soybean (73), and sorghum (74), were evaluated to identify genetic loci associated to content of up to 19 minerals.

Here, we report variation of nutrients, bioactives, and minerals of 60 distinct potato genotypes that include commonly grown cultivars and advanced breeding lines among five market classes (russets, reds, yellows, chips, and specialty potatoes). Metabolomics and ionomics was utilized to estimate and characterize the quantitative differences among potato market classes, genotypes, and variation within market class and within a genotype on potatoes suited for the commercial market. The effect on cooking was also evaluated to estimate if raw potato chemical content can predict nutrient and bioactive content of the cooked food, which would expedite screening for health-dense cultivars. Taken together, this research highlights the breadth and diversity of metabolites present in a relatively narrow potato gene pool, and lays the groundwork for future breeding efforts to generate potatoes with enhanced qualities for human health.

## Materials and methods

### Potato tuber materials for chemical analysis

Potato plants were grown in 2014 in the San Luis Valley, Colorado, USA as part of the Colorado Potato Breeding and Selection Program (http://potatoes.colostate.edu/potato-breeding/) and stored at 4°C for 3 months. A total of 60 potato genotypes were selected that span five market classes (russet, red, yellow, chip, and specialty) and represent both commonly grown cultivars and advanced breeding lines (Table 1). Potatoes were rinsed with distilled water and dried. Four potato tubers from each genotype were weighed and half (*n* = 2) were immediately frozen in liquid nitrogen while the other half (*n* = 2) were cooked via microwave as previously described (17). Briefly, the fresh weight of each potato was used to calculate cooking time, where 30 g of potato fresh weight was cooked for 1.75 min at 400 W power. After cooking, potato tubers were immediately frozen in liquid nitrogen. The cooking was chosen based on previous work that demonstrated microwaving results in less degradation of nutrients and bioactives than boiling, baking, and frying (20, 75). Raw and cooked frozen tuber samples were shattered using a hammer and freeze-dried. The freeze-dried tubers were coarsely ground with a blender followed by a fine grind in a Wiley® Mini-Mill (Thomas Scientific, Swedesboro, NJ, USA), with a 0.425 mm sieve.

**Table 1:** Potato genotypes evaluated for tuber nutrients and bioactive compounds.

**Market class**	**Breeding classification^a^**	**Cultivar/Selection**	**PVP Number**	**Female**	**Male**	**Developers^b^**	**Skin**	**Flesh**	**Usage**	**Yield potential^c^**	**References**
Chip	Cultivar	Atlantic	NA	Wauseon	Lenape	USDA-ARS	Russet	White	Chipping	Medium–high	(22, 82)
	Cultivar	Chipeta	NA	WNC612-13	Wischip	CSU, USDA-ARS, and Idaho	White	White	Chipping	High	
	Cultivar	Lenape	NA	B3672-3	47196	PSU	White	White			(83)
	Advanced line	AC00206-2W	NA	AC87340-2	Dakota Pearl	CSU and USDA-ARS	White	White	Chipping	Medium	
	Advanced line	AC01151-5W	NA	COA96142-7	NDA2031-2	CSU and USDA-ARS	White	White	Chipping	High	
	Advanced line	AC03433-1W	NA	A94322-8C	COA96141-4	CSU and USDA-ARS	White	White	Chipping	High	
	Advanced line	AC03452-2W	NA	A98423-1C	COA96141-2C	CSU and USDA-ARS	White	White	Chipping	High	
	Advanced line	AC05153-1W	NA	A91814-5	Chipeta	CSU and USDA-ARS	White	White	Chipping	Medium	
	Advanced line	CO02024-9W	NA	A91790-13W	CO95051-7W	CSU	White	White	Chipping	High	
	Advanced line	CO02033-1W	NA	A91790-13W	S440	CSU	White	White	Chipping	High	
	Advanced line	CO02321-4W	NA	NY115W	BC0894-2W	CSU	White	White	Chipping	High	
	Advanced line	CO03243-3W	NA	BC0894-2W	A91790-13W	CSU	White	White	Chipping	High	
Red	Cultivar	Colorado Rose	200500210	NDTX9-1068-11R	Cherry Red	CSU	Red	White	Fresh market	High	
	Cultivar	Rio Colorado	200800121	A8343-12	A8784-3	CSU and NDSU	Red	White	Fresh market	High	
	Cultivar	Sangre-S10	NA	Viking	A6356-9	CSU and USDA	Red	White	Fresh market	Medium-high	
	Advanced line	CO00405-1RF	NA	Banana	NDC6174-1R	CSU	Red	White	Fresh market specialty	Medium	
	Advanced line	CO00291-5R	NA	CO94019-1R	Rio Colorado	CSU	Red	White	Fresh market	Medium-high	
	Advanced line	CO00277-2R	NA	Colorado Rose	CO94065-2R	CSU	Red	White	Fresh market	High	
	Advanced line	CO05228-4R	NA	CO99256-2R	CO00292-9R						
	Advanced line	CO99256-2R	NA	Rio Colorado	Colorado Rose	CSU	Red	White	Fresh market	Very high	
	Advanced line	CO98012-5R	NA	A79543-4R	AC91844-2	CSU	Red	White	Fresh market	High	
	Advanced line	NDC081655-1R	NA	ND8555-8R	ND6126-4R						
	Advanced line	CO99076-6R	NA	AC91848-1	Rio Colorado	CSU	Red	White	Fresh market	Medium-high	
Russet	Cultivar	Canela Russet	200800122	A8343-12	A8784-3	CSU and USDA-ARS	Medium russet	White	Fresh market	Medium	
	Cultivar	Crestone Russet	201400088	AC91014-2	Silverton Russet	CSU	Medium russet	White	Dual market	High	
	Cultivar	Fortress Russet	201500349	AWN86514-2	A89384-10	CSU and USDA-ARS	Medium russet	White	Dual market	High	
	Cultivar	Mercury Russet	201400089	AC93047-1	Silverton Russet	CSU	Medium russet	White	Dual market	Medium	
	Cultivar	Mesa Russet	201200439	AO80432-1	Silverton Russet	CSU	Dark russet	White	Dual market	High	
	Cultivar	Rio Grande Russet	200500139	Butte	A8469-5	CSU and USDA-ARS	Medium-heavy russet	White	Fresh market	High	(19)
	Cultivar	Russet Burbank	NA	Early Rose	Open Pollinated		Russet	White	Fresh market	High	
	Cultivar	Russet Norkotah-S3	NA	ND9526-4Russ	ND9687-5Russ	North Dakota	Dark russet	White	Fresh market	Medium	
	Advanced line	AC00395-2RU	NA	A95523-12	Summit Russet	CSU and USDA-ARS	Medium russet	White	Dual market	High	
	Advanced line	AC96052-1RU	NA	A81386-1	GemStar Russet	CSU and USDA-ARS	Heavy russet	White	Dual market	High	
	Advanced line	CO03276-5RU	NA	CO95086-8RU	Blazer Russet	CSU	Medium-heavy russet	White	Dual market	High	
	Advanced line	CO05068-1RU	NA	AWN86514-2	CO98009-3RU	CSU	Russet	White	Dual market	High	
	Advanced line	CO05110-6RU	NA	COA96054-3	CO98009-3RU						
	Advanced line	CO05175-1RU	NA	Mesa Russet	AC96052-1RU	CSU	Russet	White	Dual market	High	
Specialty	Cultivar	Harvest Moon	201500348	Inka Gold	A89655-5DY	CSU and USDA-ARS	Purple	Yellow	Fresh market specialty	High	
	Cultivar	Mountain Rose	200500232	All Red	ND2109-7	CSU	Red	Red	Fresh market	Medium-high	(19)
	Cultivar	Purple Majesty	200500233	ND2008-2	All Blue	CSU	Purple	Dark purple	Fresh market	High	(13, 19, 21, 22, 32)
	Cultivar	Red Luna	201500350	CO94218-1	VC0967-5	CSU	Red	Yellow	Fresh market specialty	High	
	Advanced line	AC03534-2R/Y	NA	ATA98472-2Y	Mazama						
	Advanced line	AC05175-3P/Y	NA	A99331-2R/Y	COA99261-1RY						
	Advanced line	CO04063-4R/R	NA	CO97226-2R/R	CO97222-1R/R						
	Advanced line	CO04067-8R/Y	NA	CO97232-1R/Y	ATC98444-1R/Y	CSU	Red	Yellow	Fresh market specialty	High	
	Advanced line	CO05028-11P/RWP	NA	AC99329-4R/Y	CO97227-2P/PW	CSU	Red	Red, white, and purple	Fresh market specialty	High	
	Advanced line	CO05028-4P/WPY	NA	AC99329-4R/Y	CO97227-2P/PW						
	Advanced line	CO05037-2R/Y	NA	Harvest Moon	CO97227-2P/PW	CSU	Red	Yellow	Fresh market specialty	Medium	
	Advanced line	CO05079-4P/PW	NA	CO97216-3P/PW	CO97227-2P/PW						
	Advanced line	CO97226-2R/R	NA	Mountain Rose	CO94214-1	CSU	Red	Red	Fresh market specialty	Medium	
	Advanced line	CO97227-2P/PW	NA	Mountain Rose	CO94215-1	CSU	Purple	Purple	Fresh market specialty	High	
Yellow	Cultivar	Masquerade	201400086	Inka Gold	A91846-5R	CSU and USDA-ARS	Purple and white bicolor	Yellow	Fresh market specialty	High	
	Cultivar	Yukon Gold	NA	Norgleam	W5279-4	Canada	White	Yellow	Fresh market	Medium	(22, 84)
	Advanced line	ATC00293-1W/Y	NA	Agria	TXA1655-1DY	CSU, USDA-ARS, and Texas A&M	White	Yellow	Fresh market specialty	High	
	Advanced line	CO00412-5W/Y	NA	German Butterball	TX1523-1RU/Y	CSU	White	Yellow	Fresh market specialty	High	
	Advanced line	CO04099-3W/Y	NA	VC1002-3W/Y	ATC98495-1W/Y	CSU	white	Yellow	Fresh market specialty	High	
	Advanced line	CO05035-1PW/Y	NA	Masquerade	US147-96						
	Advanced line	CO05037-3W/Y	NA	Midnight Moon	CO97227-2P/PW	CSU	White	Yellow	Fresh market specialty	High	
	Advanced line	CO07131-1W/Y	NA	PA4X137-12	4X91E22	CSU	White	Dark yellow	Fresh market	Low	
	Advanced line	CO99045-1W/Y	NA	Rio Grande Russet	German Butterball	CSU	White	Yellow	Fresh market specialty	Very high	

a *Breeding classification: Cultivar (named and released cultivar); advanced line (at least 6 years of selection, not released)*.

b *Developers: CSU, Colorado State University; USDA-ARS, United States Department of Agriculture-Agricultural Research Services; PSU, Pennsylvania State University; NDSU, North Dakota State University*.

c *Yield potential for San Luis Valley Research area: very low, ≤ 250 hundredweight/acre (cwt/A); low, 251–300 cwt/A; medium, 301–350 cwt/A; medium-high, 351–400 cwt/A; high, 450–500 cwt/A; very-high, >501 cwt/A*.

### Metabolite extraction

A biphasic extraction was utilized to optimize the extraction of a wide range of chemical compounds (62, 76, 77). One milliliter of a cold (−20°C) biphasic solution [6:3:1; Methyl tert-butyl ether (MTBE): Methanol (MeOH): Water; v:v:v] was added to 100 mg of freeze-dried potato tuber powder in an ice bath, and the samples were vortexed at 4°C for 1 h. After 1 h, 750 μL of cold (4°C) water was added to induce phase separation. Samples were centrifuged for 25 min at 2,850 x g at 4°C. The upper (organic) and lower (aqueous) phases were collected and placed in separate 2 mL glass vials and stored at −20°C.

### Metabolite detection using ultra performance liquid chromatography mass spectrometry (UPLC-MS)

For UPLC-MS metabolite analysis, the organic and aqueous extracts were combined (2:1, v:v) and dried using a speedvac. UPLC-MS analysis of the aqueous (Supplementary Figure 1A), organic (Supplementary Figure 1B), and a combination of aqueous and organic (Supplementary Figure 1C) revealed that recombining the aqueous and organic fractions in a 2:1 (v:v) ratio resulted in much broader coverage of the potato metabolome. Samples were re-suspended in 100 μL of MTBE: MeOH: Water (6:3:1, v:v:v) and 5 μL of metabolite extract was injected into an Acquity UPLC system (Waters Corporation). Metabolite separation and detection methods were performed as previously described (78). Separation was performed using an Acquity UPLC CSH Phenyl-Hexyl column (1.7 μm, 1.0 × 50 mm; Waters Co.), at a constant flow rate of 200 μL/min, using a gradient from solvent A (2 mM of ammonium hydroxide, 0.1% formic acid) to solvent B (acetonitrile, 0.1% formic acid). Injections were made in 100% A and held for 1 min, a 13 min linear gradient to 95% B was then applied, and held at 95% B for 3 min. The system was returned to starting conditions over 0.05 min and allowed to re-equilibrate for 3.95 min. The column was held at 65°C while samples were held at 6°C. The column eluent was infused into a Waters G2 ESI-TOF mass spectrometer with an electrospray ion source acquiring in positive ion mode scanning 50–1,200 m/z at 0.2 s per scan, alternating between MS (6 V collision energy) and MS^E^ mode (15–30 V ramp). Sodium iodide was used for calibration with 1 ppm mass accuracy. The capillary voltage was held at 2,200 V, source temp at 150°C, and nitrogen desolvation temperature at 350°C with a flow rate of 800 L/h. Replicate injections of each sample were used as quality control to account for analytical variation.

### Metabolite detection using gas chromatography mass spectrometry (GC-MS)

GC-MS analysis was performed on the aqueous phase of the potato extract. Initial analyses demonstrated the need to utilize different volumes of the aqueous layer for cooked and raw potato due to the GC-MS inlet, column, and/or detector saturating due to excess saccharides in cooked potato. Specifically, 150 μL (raw potato) or 75 μL (cooked) of aqueous phase extract was transferred to a new tube and dried using a speedvac. Derivatization (methoximation and silylation) and GC-MS detection was performed as previously described (78). Briefly, 50 μL of pyridine containing 15 mg/mL of methoxyamine hydrochloride was added and samples were incubated for 45 min at 60°C, sonicated for 10 min, and incubated again for 45 min at 60°C. Subsequently, 50 μL of N-Methyl-M-(trimethylsilyl) trifluoroacetamide (MSTFA) + 1% trimethylchlorosilane (TMCS) (ThermoFisher Scientific, Waltham, MA, USA) was added and samples were incubated at 60°C for 30 min. Samples were centrifuged at 2,850 x g at 4°C, and 80 μL of the supernatant was transferred to a 150 μL glass insert. GC-MS was performed using a Trace GC Ultra coupled to a Thermo DSQ II (Thermo Scientific, Waltham, MA, USA. Metabolites were separated with a 30 m TG-5MS column (Thermo Scientific, 0.25 mm i.d. 0.25 μm film thickness). The program began at 80°C for 30 s, ramped to 330°C at a rate of 15°C per min, and ended with an 8 min hold at a 1.2 mL/min helium gas flow rate. Masses between 50 and 650 m/z were scanned at five scans/s after electron impact ionization. The inlet temperature was held at 280°C and the auxiliary line was held at 300°C. Replicate injections of each sample were used as quality control to account for analytical variation.

### Metabolomics data processing

UPLC- and GC-MS files were converted to .cdf format and each set was independently processed by XCMS (79, 80) in R (81). Samples were normalized to total ion current and relative abundance for each molecular feature was determined by the mean area of the chromatographic peaks among replicate injections (*n* = 2). UPLC- and GC-MS data were deconvoluted into spectral clusters using RAMClust (85). Metabolites were identified by matching mass spectra and retention indices and/or experimental or predicted retention times with in-house and external databases (86) including NIST (http://nist.gov), Golm Metabolome Database (87, 88), Lipid Maps (89), and Human Metabolome database (90). Confidence in metabolite annotations was based on guidelines of the Metabolomics Standards Initiative (91). UPLC may sometimes separate metabolite isomers, and these are observed as identical mass spectra at different retention times. Metabolite isomers are indicated as numbers next to metabolite names (“metabolite 01,” “metabolite 02,” etc.).

### Sample preparation and acid digestion for ionomics analysis of raw potato tubers

A total of 150 mg of raw freeze-dried potato tuber powder was added to a 16 × 110 mm borosilicate glass test tube. Subsequently, 1.5 mL of 70% nitric acid (BDH Aristar® Plus) was added followed by 66.7 μL of internal standard solution [10 ppm each of Bismuth (Bi), Gallium (Ga), Indium (In), Scandium (Sc), and Yttrium (Y)]. Samples were gently mixed, covered with plastic wrap, and digested overnight at room temperature. Next, samples were heated in a sand bath for 3 h at 120°C, cooled at room temperature for 5 min, and then 750 μL of hydrogen peroxide (J.T. Baker, 30% Ultrex® II Ultrapure reagent) was added to each sample. The solution was heated in a sand bath at 120°C for an additional hour. Samples were removed from the sand bath and allowed to cool to room temperature. The digest was transferred to 15 mL centrifuge tube and diluted to 10 mL using ultrapure 18.2 MΩ water, and 4.5 mL of the diluted solution was transferred to a new 15 mL centrifuge tube. The solution was subsequently diluted to a final volume of 15 mL using ultrapure water. The final solution contained internal standard concentrations of 20 ppb in 3% nitric acid.

### Ionome detection using inductively coupled plasma mass spectrometry (ICP-MS)

Elemental concentrations of Arsenic (As), Aluminum (Al), Barium (Ba), Boron (B), Beryllium (Be), Cadmium (Cd), Calcium (Ca), Chromium (Cr), Cobalt (Co), Copper (Cu), Iron (Fe), Lead (Pb), Lithium (Li), Magnesium (Mg), Manganese (Mn), Molybdenum (Mo), Nickel (Ni), Phosphorous (P), Potassium (K), Selenium (Se), Sodium (Na), Strontium (Sr), Sulfur (S), Vanadium (V), Tungsten (W), and Zinc (Zn) were measured using an Elan DRC (Dynamic Reaction Cell) II mass spectrometer (PerkinElmer, Akron, OH, USA) connected to a Seaspray™ MEINHARD nebulizer and a quartz cyclonic spray chamber. Samples were introduced using an ASX-520 autosampler (CETAC Technologies, Omaha, NE, USA). Li, Be, B, Na, P, S, Mg, K, Ca, W, and Pb were measured in standard mode. To reduce polyatomic interferences, some elements were measured in DRC mode. Cd, Se, and As were measured in DRC mode using oxygen as the reactive gas. Al, V, Cr, Mn, Fe, Co, Ni, Cu, Zn, Sr, Mo, and Ba were measured in DRC mode using ammonia as the reactive gas. Before analysis, the nebulizer gas flow and lens voltage were optimized for maximum Indium signal intensity (56,008 counts per second), with final values of 0.85 (L/min) and 8.0, respectively. A daily performance check was also run which ensured that the instrument was operating properly and obtained a CeO^+^:Ce^+^ of 0.028 and a Ba^++^:Ba of 0.017. A calibration curve was obtained by analyzing seven dilutions of a multi-element stock solution made from a mixture of single-element stock standards (Inorganic Ventures, Christiansburg, VA, USA). To correct for instrument drift a quality control (QC) solution (pooled sample, prepared by mixing 2 mL of each digested individual sample) was run every 10^th^ sample.

### Ionomics data processing

Data was processed using Microsoft® Excel. Each element was subjected to internal standard corrections and subsequently drift corrected (92). Corrections were chosen based on minimizing the relative standard deviation (RSD) for the QC samples. After drift correction, samples were corrected for the dilution factor. Limits of detection (LOD) and limits of quantification (LOQ) were calculated as 3 times or 10 times the standard deviation of the blank divided by the slope of the calibration curve, respectively (93, 94). Final concentrations are reported as ppb (μg/kg of freeze-dried potato). Measured calculations below the LOQ were assigned to the LOQ/2 (95).

### Statistical analysis

For metabolites, Spearman's correlations and hierarchical clustering were conducted in R (81) using corr and hclust functions, respectively. Metabolites and elements were evaluated using analysis of variance (ANOVA) using the aov function in R (81). For ANOVA, a *p* threshold of 0.05 was used following a Benjamini-Hochberg (96) adjustment using the p.adjust function in R (False Discovery Rate, FDR). Principal component analysis (PCA) of metabolites and elements was performed on mean-centered and unit variance scaled data using SIMCA v14.1 (Umetrics, Umea, Sweden). Z scores for metabolites were calculated using the relative abundance value of a metabolite compared to the mean and standard deviation of the metabolite's relative abundance across all samples (i.e., the population mean and standard deviation). Z scores were used to generate a heat map using the heatmap.2 function of gplots package in R (97). Fold variation (FV) for cooked nutrient and bioactive compounds was calculated within and among market classes. Within market class mean FV (mFV) was calculated as ratio of the potato genotype with the highest metabolite mean peak area (*n* = 2) divided by the potato genotype with the lowest mean peak area (*n* = 2) within each potato market class for each nutrient and bioactive compound (Table 2). Among market classes mFV was calculated as the ratio of the highest average mean metabolite peak area of the potato market class divided by the lowest average mean peak area potato market class for each nutrient and bioactive compound (Table 2). Relative standard deviation (RSD) was determined for cooked and raw metabolites and between market classes within raw and cooked tubers. The RSD was calculated for each individual cultivar and then averaged (via mean) across treatments and represented as a heat map using the heatmap.2 function in gplots (97). Spearman's rank correlations of cooked vs. raw metabolites was visualized using the corrplot package in R (111).

**Table 2:** Mean fold variation of bioactive and nutrient compounds identified in potato tubers.

**Category**	**Class**	**Annotation**	**Mean fold variation**^**a**^						**Health effects**	**Selected publications**
			**Cooked**							
			**Market class^b^**	**Cultivar**^c^						
				**Chip**	**Red**	**Russet**	**Yellow**	**Specialty**		
Bioactives	Alkaloids	Calystegine A3	2	8	28	20	10	21	Treat diabetes	(38)
		Calystegine B2	9	29	26	742	34	626	Treat diabetes	(38)
		Trigonelline	2	4	10	3	68	5	Hypoglycemic activity, neuroprotective	(39)
	Amides	Oleamide 01	2	5	5	3	4	8	Vasorelaxant	(35)
		Oleamide 02	1	3	3	3	4	5		
	Amines/Polyamines	Kukoamine 01	6	13	173	5	8	23	Vasorelaxant	(36)
		Kukoamine 02	6	13	88	3	7	19		
		Kukoamine 03	6	14	214	3	6	21		
	Coumarins	4-Methylumbelliferone	2	5	2	4	2	2	Anticancer, antiproliferative activity	(98)
	Flavonoids	Apigenin	1	4	3	3	3	4	Anticancer, antiproliferative activity	(29)
		Kaempferol 3-O-rutinoside	48	14	15	13	9	636	Hypotensive activity, antihypertensive activity	(33)
		Rutin	4	38	39	64	37	21	Anticancer, inhibition of tumor growth	(99)
	Glycoalkaloids	α-Solamarine 01	2	7	6	9	3	14		
		α-Solamarine 02	2	10	4	15	9	14		
		β-Chaconine conjugate	4	5	7	12	9	304		
		Chaconine 01	2	3	2	6	3	3	Anticancer, inhibition of tumor angiogenesis	(23, 30)
		Chaconine 02	1	2	2	4	2	3		
		Chaconine 03	1	5	4	9	3	5		
		Chaconine 04	2	4	3	11	4	4		
		Solanine 01	3	7	9	42	61	8	Anticancer	(100)
		Solanine 02	8	4	62	45	155	66		
		Solanine 03	1	2	2	4	3	3		
		Solanine 04	1	3	3	6	3	3		
		Solanine 05	2	4	7	45	7	7		
		Solanine 06	2	3	2	5	3	3		
		Solanine 07	2	7	3	26	3	8		
		Solanine 08	3	105	74	493	51	79		
		Solanine 09	2	8	7	8	19	5		
		Solanine 10	2	20	5	10	3	8		
		Solanine-like	1	4	2	3	2	3		
		Tomatine	2	11	12	12	18	46	Cholesterol lowering	(52)
	Phenolics	Chlorogenic acid 01	3	3	2	3	2	9	Hypotensive activity	(37)
		Chlorogenic acid 02	7	6	3	9	5	30		
		Chlorogenic acid 03	40	16	9	43	24	526		
		Isoferulic acid	2	4	5	3	5	4	Hypoglycemic activity	(101)
		Quinic acid 01	18	11	9	10	14	157	Antiviral activity	(102)
		Quinic acid 02	2	4	7	17	9	16		
	Purines	Adenosine	2	4	2	4	4	3		
	Saccharides	Saccharide	2	3	2	3	4	7		
		Threonic acid	1	3	4	15	4	7		
	Xanthophylls	Lutein	4	2	2	3	14	4	Photoprotectant	(103, 104)
		Neoxanthin 01	1	11	4	4	2	4	Anti-obesity	(46)
		Neoxanthin 02	13	2	2	2	7	14		
Nutrients	Amino acids	Alanine	2	7	23	8	5	5		
		Asparagine 01	3	24	7	24	4	12		
		Asparagine 02	2	8	14	18	11	7		
		Aspartic acid 01	2	3	18	6	4	8		
		Aspartic acid 02	2	2	1	2	2	4		
		β-Alanine	2	7	9	9	7	9		
		Glutamate	4	54	35	73	53	111		
		Glutamine 01	1	3	2	2	2	2		
		Glutamine 02	3	20	3	11	3	2		
		Glutamine 03	3	103	23	5	5	16		
		Glutamine 04	4	288	467	69	70	249		
		Glutamine 05	2	6	2	3	6	4		
		Glycine	2	5	8	5	5	2		
		Isoleucine 01	4	9	16	11	16	14		
		Isoleucine 02	4	5	31	7	4	8		
		Leucine	3	5	30	5	5	10		
		Methionine	1	5	4	6	5	4		
		Phenylalanine 01	2	3	2	2	2	5		
		Phenylalanine 02	4	9	29	15	13	78		
		Phenylalanine 03	2	11	8	7	3	32		
		Proline	3	5	37	86	28	4		
		Serine 01	3	4	21	11	6	9		
		Serine 02	1	10	7	9	5	5		
		Serine 03	2	6	7	10	7	5		
		Threonine 01	3	4	6	5	3	4		
		Threonine 02	3	5	24	15	13	8		
		Tryptophan 01	2	4	2	4	3	4		
		Tryptophan 02	5	18	9	15	32	57		
		Tyrosine	3	27	7	6	8	6		
		Valine 01	6	16	39	13	5	5		
		Valine 02	3	6	39	5	5	3		
	Fatty acids	Linolenic acid	1	2	2	2	2	3		(105)
	Organic acids	Citric acid	2	2	2	2	2	3		
	Saccharides	Mannose	3	84	248	231	8	70		
	Vitamins	α-Tocopherol 01	1	2	2	2	2	2		(106)
		α-Tocopherol 02	1	2	4	3	2	5		
		γ-Tocopherol 01	2	3	2	4	3	2		(107)
		γ-Tocopherol 02	3	34	14	21	16	23		
		Pantothenic acid 01	1	2	2	2	2	2		(108)
		Pantothenic acid 02	1	2	2	2	2	2		
		Pyridoxal	2	3	3	2	3	6		(109)
		Pyridoxine	1	1	2	3	2	2		(110)

a *Within market class mean Fold Variation = (potato genotype with the highest metabolite mean peak area)/(potato genotype with the lowest mean peak area). Among market class mean Fold Variation = (potato market class with highest metabolite mean peak area)/(potato market class with the lowest metabolite mean peak area)*.

b *Among market class mean Fold Variation*.

c *Within market class mean Fold Variation*.

## Results

### Metabolomics detected and quantified a diverse set of bioactives and nutrients in potato tuber

Non-targeted UPLC- and GC-MS metabolomics was conducted on 60 potato genotypes that span 5 market classes: russet, red, chip, yellow, and specialty (Table 1). The population was developed to characterize tuber chemical diversity with diverse genetics, however all samples would be considered acceptable in the consumer market. The sample set included released cultivars and advanced breeding lines, as well as several levels of maturation, yield potential, and a diverse breeding pedigree as the basis for genotypic diversity.

The UPLC- and GC-MS analyses detected 1,757 and 899 compounds, respectively, for a total of 2,656 compounds. Of the 2,656, 185 were annotated as a known metabolite, 42 are known nutrients, and 43 were classified as a bioactive compound (“bioactives”; Table 2). The bioactives included several types of alkaloids, amides, amines, polyamines, phenolics (coumarins, flavonoids), and terpenes (carotenoids). The nutrients included amino acids, fatty acids, organic acids, saccharides, and vitamins (Table 2). An additional 76 compounds were classified as lipids and 24 were classified as “other.” Further, the potato metabolome contained many essential nutrients such as amino acids (isoleucine, leucine, lysine, methionine, phenylalanine, threonine, tryptophan, and valine). We were also able to detect the essential fatty acid linolenic acid, and essential vitamins such as vitamin E (α- and γ-tocopherol), vitamin B5 (pantothenic acid), vitamin B6 (pyridoxal and pyridoxine) (Table 2).

### Cooking influences the potato tuber metabolome resulting in the reduction of many bioactives and nutrients

The metabolite profiles of cooked and raw potato tuber were compared to understand (i) which nutrients and bioactives are sensitive to high temperatures and are reduced during cooking and (ii) if potato genotypes with the highest levels of nutrients and bioactives in raw tuber are also highest in cooked tuber. These data are important to understand the potential to screen potato tuber to identify unique genotypes with superior health properties.

**Figure 1:**
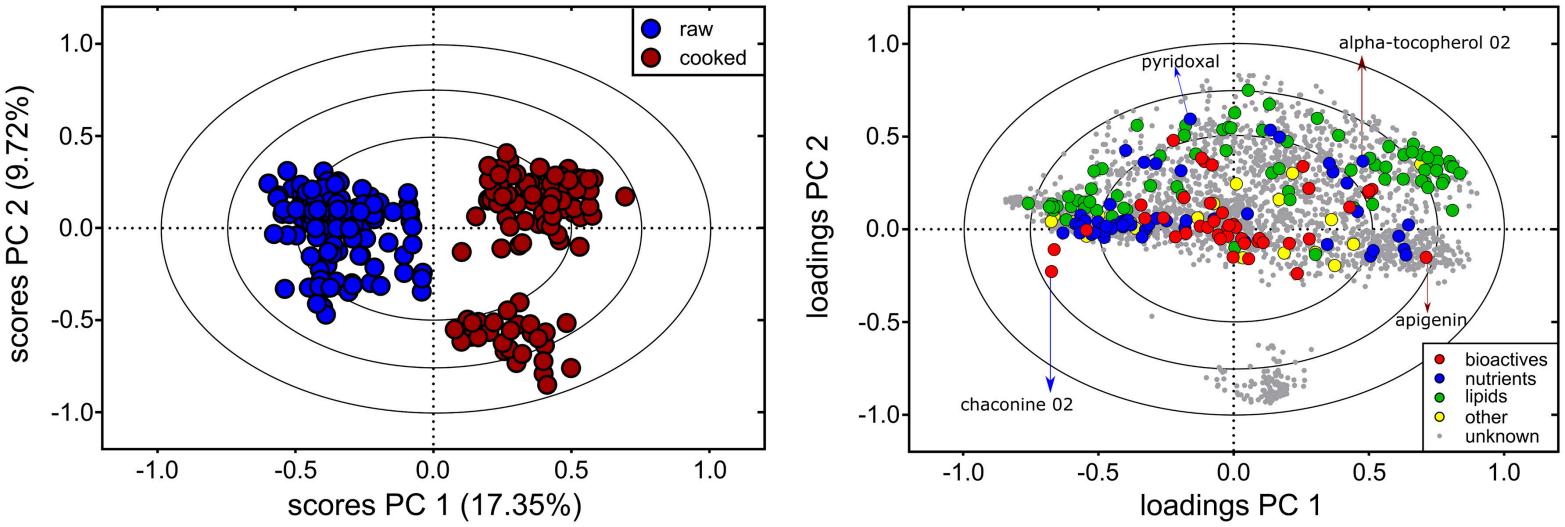
Influence of cooking on the potato tuber metabolome. Principal Component Analysis (PCA) of the 60 cooked (red) and raw (blue) potato tuber metabolomes show that cooking was a major facet of metabolite variation, indicated by separation along PC 1 in the scores plot (left). Metabolite variation attributed to other factors was also observed (i.e., PC 2). The 2,656 metabolites detected are shown on the PC loadings plot (right), and colors represents metabolite class (bioactives, nutrients, lipids, others, and unknowns). Example nutrients and bioactives are indicated by arrows. Red arrows indicate nutrients or bioactives more abundant in cooked potato tubers and blue arrows indicate nutrients or bioactives more abundant in raw potato tubers. The PCA loadings and scores plot are correlation scaled and ellipses denote 0.5, 0.75, and 1.0 correlation values.

**Figure 2:**
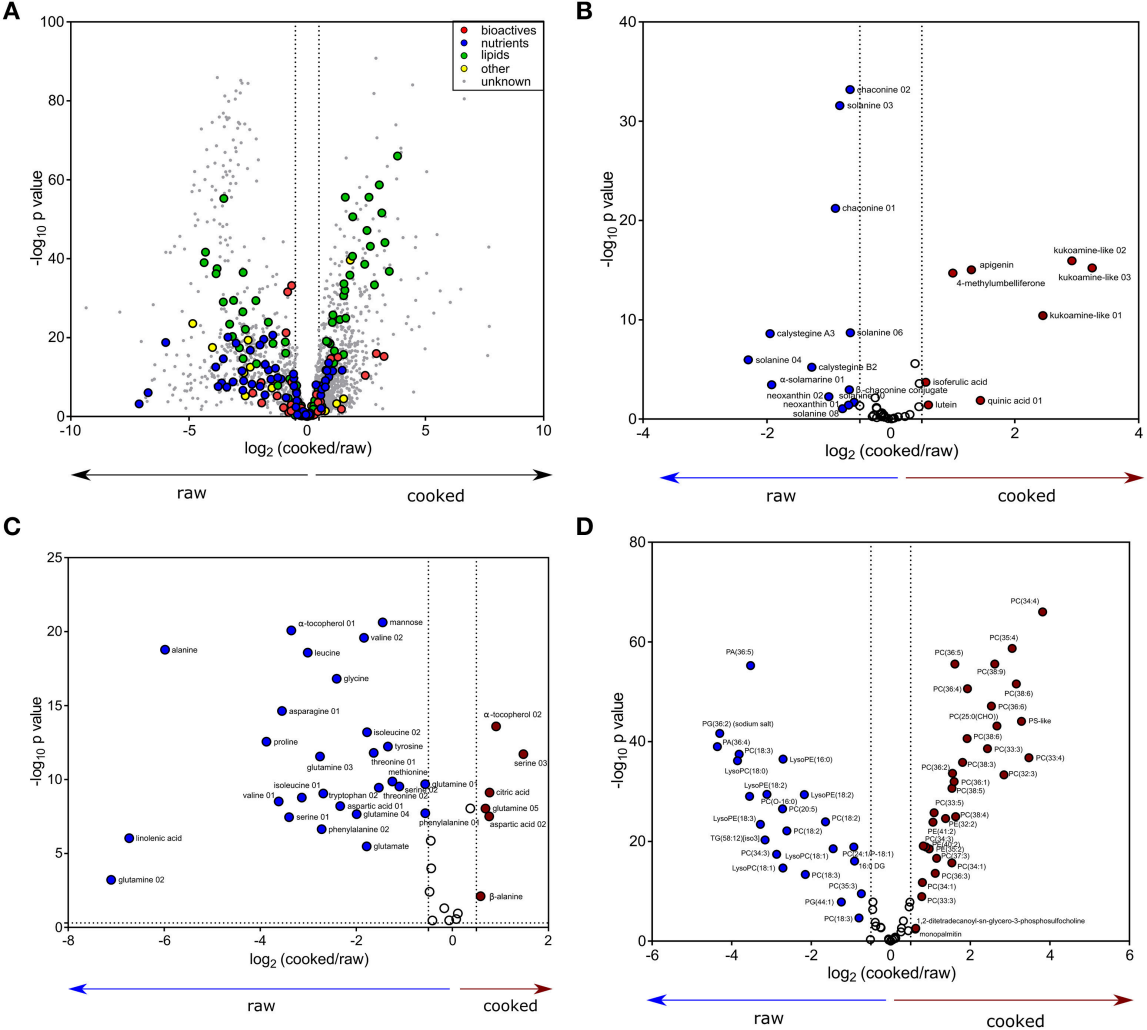
Variation in cooked and raw potato tuber metabolites. **(A)** Volcano plot for the differential abundance (log_2_ cooked/raw, x-axis) and significance (–log_10_ FDR adjusted *p*-value, y-axis) of 2,656 metabolites (colored circles) detected by UPLC- and GC-MS across 60 cultivars of raw and cooked potato tubers. Color represents metabolite class, and vertical dashed lines are a threshold of cooked/raw [log_2_(cooked/raw) < -0.5 or >0.5]. Subsets of the volanco plot in **(A)** were recreated for **(B)** bioactives, **(C)** nutrients, and **(D)** lipids. The subset volcano plots are colored to indicate metabolites reduced during cooking (blue), increased during cooking (red), or metabolites that did not vary due to cooking (white).

Metabolite profiles of the 60 genotypes (cooked and raw) were evaluated using PCA (Figure 1, left; PC 1, 17.35% of the variation). Overall, 1,977 out of the 2,656 detected compounds (74.4%) varied due to cooking (ANOVA, FDR adjusted *p* < 0.05). Cooking influenced the abundance of 24/43 bioactives (55.8%) and 37/42 nutrients (88.1%) (ANOVA, FDR adjusted *p* < 0.05, Supplementary Table 1). The PCA loadings plot indicates no major trend in all bioactives, nutrients, lipids (e.g., some nutrients increased, and others decreased; Figure 1). The effects of cooking were further evaluated with a fold variation analysis (log_2_ cut-off of ± 0.5) and show that 41% (1,102 of 2,656) of the metabolites did not vary with cooking, 33% (864 of 2,656) were more abundant in cooked potato, and 26% (690 of 2,656) were more abundant in raw potato (Figure 2A). Several bioactives decreased following cooking including neoxanthin, glykoalkaloids (α-solamarine, solanine, chaconine, and some of their isomers), calystegine A3 and calystegine B2 (Figure 2B). Nutrients that decreased following cooking include most amino acids and linolenic acid (Figure 2C). Importantly, several bioactives and nutrients did not vary with cooking such as chlorogenic acid, tomatine, rutin, oleamide, trigonelline, pantothenic acid, pyridoxal, and γ-tocopherol (Figures 2B,C). Further, multiple lipids were observed to vary with cooking, however there was no major trend of increasing or decreasing in abundance according to lipid chemical class (Figure 2D).

### Market class had a minor influence on the abundance of nutrients and bioactives

**Figure 3:**
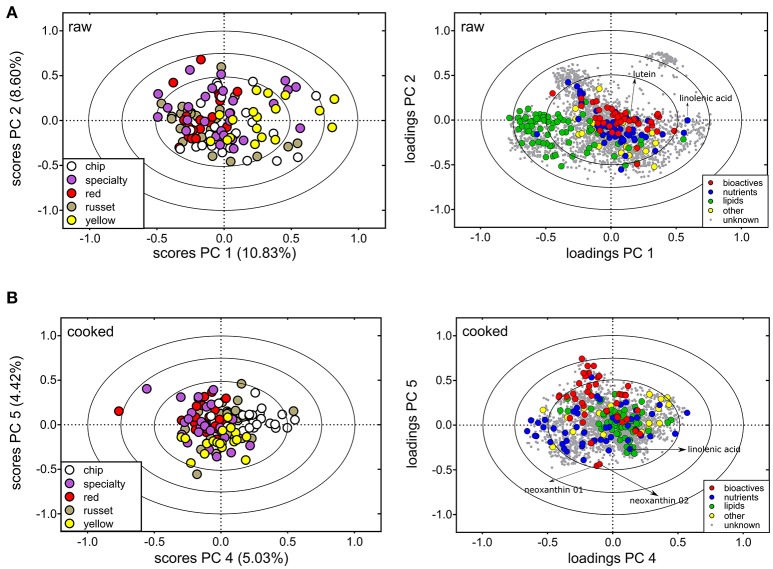
Metabolite variation among potato market classes. PCA analysis for **(A)** raw tuber and **(B)** cooked tuber colored according to potato market class. For raw potato, PC 1 and PC 2 explained variation among market class for 124 metabolites (colored circles). For cooked potato, PC 4 and PC 5 separated market class explained by 100 metabolites. Metabolites denoted on the PCA loadings plot exhibit increased abundance in yellow potato. PCA loadings and scores plot are correlation scaled and ellipses denote 0.5, 0.75, and 1.0 correlation values.

Analysis of the metabolites detected in both raw and cooked potato reveal market class differences (Figures 3A,B), however, there was more metabolite variation within market classes than among market class (Table 2, Supplementary Table 1). The PC scores plots within raw potato showed most separation was due to the yellow market class along PC 1 (10.83% of the variation). These potatoes have high carotenoid content resulting in yellow/orange internal flesh (e.g., the cultivar Yukon Gold) (112–115), and, not surprisingly, carotenoids were major contributors to the separation of the yellow market class from others (e.g., lutein, Figure 3A, right). Furthermore, the fatty acid linolenic acid was also more abundant in yellow potatoes (Figure 3A, right). Interestingly, the raw yellow potatoes had overall reduced levels of lipids compared to all other market classes (Figure 3A, right). The metabolite variation among market classes, specifically differences between yellow potatoes and all others, did exist for cooked potato but to a lesser extent (Figure 3B). Variation among market classes was observed via PC 4 of the analysis (5.03% of the variation; Figure 3B). The major contributor to the market class separation was the chip class, which has little nutritional relevance as these potatoes are rarely consumed in the fresh market. Separation of yellow potatoes is observed along PC 5 (4.42% of the variation, Figure 3B), with linolenic acid and xanthophylls (neoxanthin and lutein) primarily contributing to this separation (Figure 3B, right).

### Metabolite co-variation analysis supports the potential to breed for genotypes with high levels of bioactives and nutrients in cooked tuber

**Figure 4:**
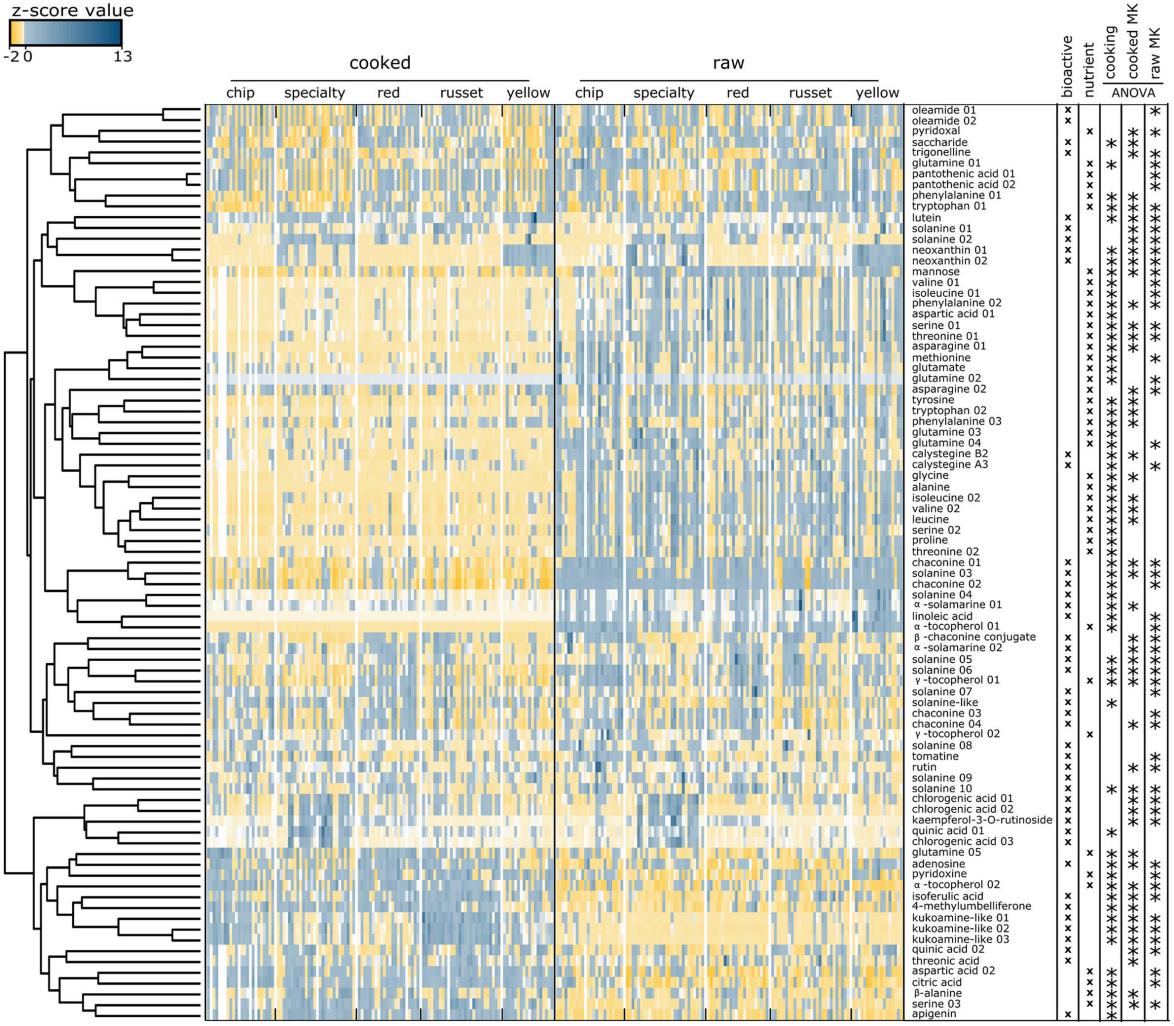
Heat map of nutrients and bioactive compounds identified in cooked and raw potato tubers. Z-score values of the 85 metabolites annotated as nutrients or bioactives evaluated by hierarchical clustering. Two main clusters were formed: metabolites in high abundance in raw tubers and low abundance in cooked tubers (top), and metabolites in low abundance in raw and high abundance in cooked (bottom). Each colored square represents the centered and scaled relative abundance of a metabolite (z-score). Z-scores were calculated as follows: z = (X – μ)/σ, where X is the relative abundance of a metabolite, μ is the mean abundance for the metabolite among all samples, σ is the standard deviation among all samples. Hierarchical clustering was performed using Euclidian distances. Metabolite names with a number indicate isomers of the same compound. Statistical significance was calculated using a one-way ANOVA and adjusted for false discovery.

The co-variation of 85 metabolites was evaluated among market classes, genotypes, and cooking by integrating a z transformation of the metabolite abundances with hierarchical clustering visualized as a heat map (Figure 4). This result further supports that sets of nutrients/bioactives were (i) higher in raw tuber, (ii) higher in cooked tuber, or (iii) were not influenced by cooking. For example, a coumarin, 4-methylumbelliferone, isoferulic acid, and apigenin were more abundant in cooked tuber, whereas α-solamarine and linolenic acid were more abundant in raw tuber. Chlorogenic acid, kaempferol-3-O-rutinoside, quinic acid, and oleamide were not influenced by cooking.

The heat map further demonstrates differences between potato market classes. The xanthophyll neoxanthin was more abundant in both raw and cooked yellow potatoes (Figure 4) and linoleic acid was more abundant in raw yellow potatoes as compared to other market classes. Specialty raw potatoes had more chlorogenic acid, kaempferol-3-O-rutinoside, and quinic acid when compared to russet, chip, yellow, and red potatoes. Vitamin B5 (pantothenic acid) was less abundant in specialty potato and the alkaloid trigonelline was less abundant in red potatoes. Cooked russets and chips had the most kukoamine compared to red, yellow, and specialty potatoes (Figure 4).

### Increased metabolite variation was observed within market classes than among market class

Fold Variation (FV) analysis of cooked nutrient and bioactive compounds revealed more variation within market class than among market class (Table 2). The extent of variation within market class was defined by a mean FV (mFV) of 30 bioactives or 19 nutrients. The data further support that most mean nutrient and metabolite abundances did not vary among market class. For the entire potato population, there was a mFV of 4 for all nutrients and bioactives. In general, bioactives exhibited greater variation in the full potato population (mFV of 43 bioactives = 5) compared to nutrients (mFV of 42 nutrients = 2). However, market classes differed more broadly in terms of the range of variation for bioactives and nutrients. Specialty potatoes exhibited the greatest variation for all metabolites (mFV = 42), and yellow potatoes had the lowest total variation (mFV = 12). For bioactives, specialties had the most variation (mFV = 64) and chips had the least variability (mFV = 10). For nutrients, reds had the highest variation (mFV = 29) and yellows had the least (mFV = 9).

**Figure 5:**
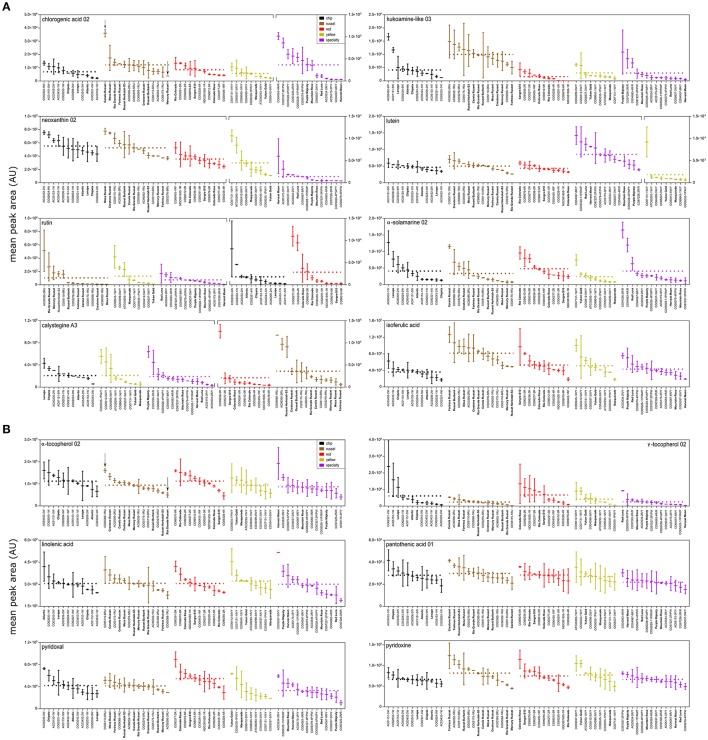
Metabolite variation among and within potato market class. Box and whisker plots of **(A)** bioactive and **(B)** nutrient compounds in cooked potato demonstrate greater variation within potato market class than among potato market class. Potato genotypes within each market class (colored) are arranged from highest mean peak area (normalized abundance) to lowest mean peak area. Fold variation within a market class is calculated as mean normalized abundance of the highest potato cultivar within a potato market class divided by the mean normalized abundance a of the lowest potato variety within a potato market class. Dashed line represents mean normalized abundance for a market class. Fold variation among potato market classes is calculated as mean normalized abundance of the highest potato market class over average mean peak area of lowest potato market class. Potato genotypes in bold denote potato cultivars (released commercial varieties). Plot breaks are used to account for plotting large differences in metabolite abundances.

The variation of important bioactive and nutrient compounds was also visualized as box plots (Figure 5). Three key trends were observed in the data: (i) there were minimal differences in mean metabolite abundance among market classes; (ii) metabolites were normally distributed within market classes; and in some instances; (iii) select potato genotypes had significantly more of a compound than most. For example, chlorogenic acid displayed minor overall variation among market classes, exhibited a normal distribution within a market class, and one russet (Canela Russet) and several specialty genotypes (CO04063-4R/R and CO97227-2P/PW) had significantly more chlorogenic acid than most genotypes (Figure 5A). Similar trends were observed for other bioactives (Figure 5A). Some compounds were more abundant among market classes, such as lutein and neoxanthin, however these were also highly abundant in specialty potatoes that have been developed to also have yellow internal flesh (Harvest Moon, Red Luna, AC03534-2R/Y, AC05175-3P/Y, CO04067-8R/Y, and CO05037-2R/Y; Figure 5A). Interestingly, the alkaloid calystegeine A3, was overall more abundant in the russet market class, but a specific red potato, CO99256-2R has significantly greater quantities (Figure 5A) than all russet potatoes. Similar trends are observed for bioactive compounds such as rutin, α-solamarine, and isoferulic acid (Figure 5).

Nutrient compounds also demonstrated similar patterns (Figure 5B). Overall, vitamins (α-tocopherol, γ-tocopherol, pantothenic acid, pyridoxal, and pyridoxine) did not vary among market classes and had a normalized distribution within market class. Linolenic acid also exhibited the same pattern where a single specialty potato (CO05037-3WY) had significantly more linolenic acid than most potato genotypes (Figure 5B). Overall, nutrient compounds were stable across genotypes except for linolenic acid.

### Bioactives and nutrients found in raw potato tubers correlate with cooked potato tubers

**Figure 6:**
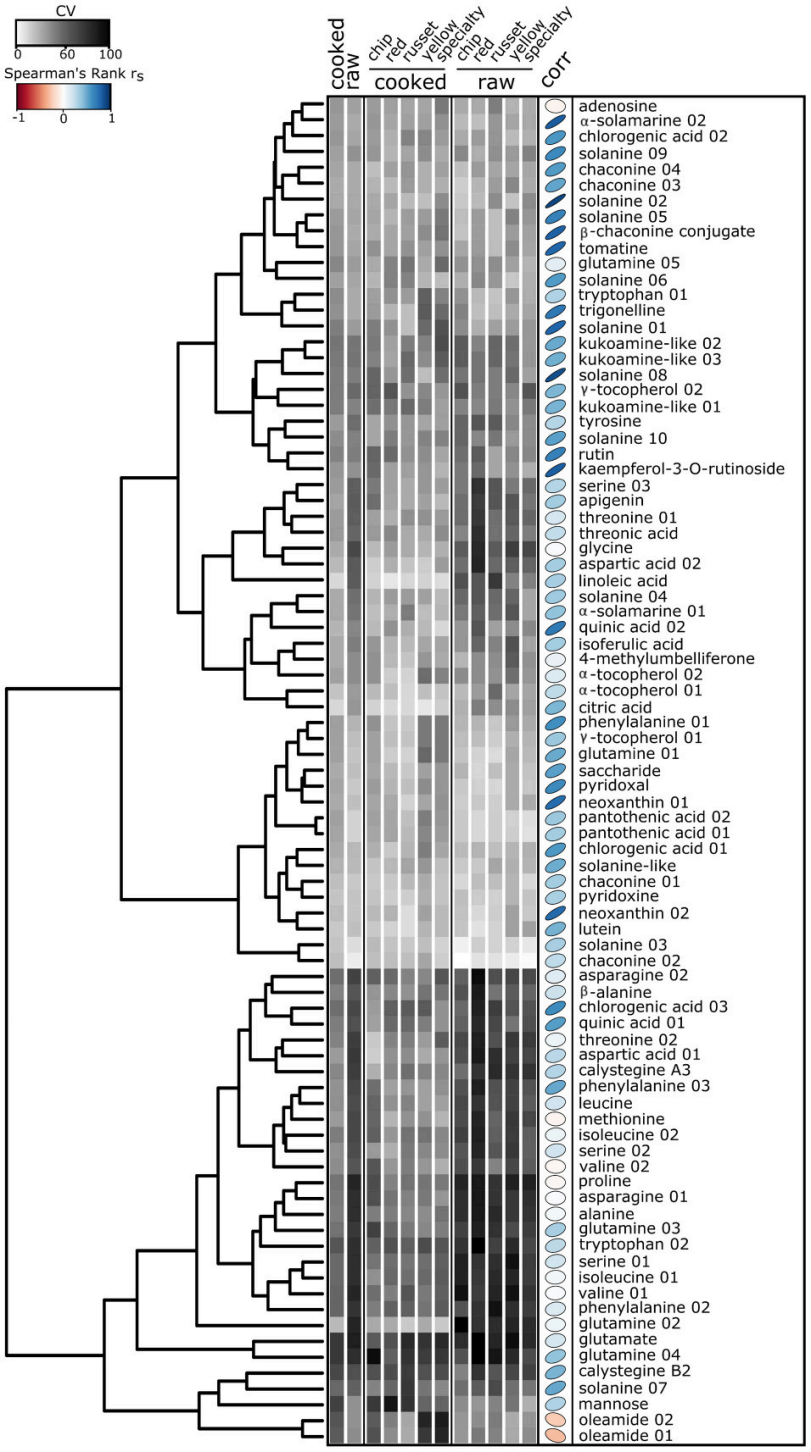
Heat map of the relative standard deviation of nutrients and bioactive compounds identified in cooked and raw potato tubers. Gray squares represent the mean coefficient of variation (CV) for each of the 85 nutrients and bioactives within raw or cooked samples (*n* = 120), and among market class within raw or cooked tubers. CV was calculated as: CV = σ/μ ^*^100, where σ is the standard deviation or the metabolite for each individual cultivar within a treatment and μ is the mean abundance for the metabolite for each individual cultivar within a treatment. The CV is calculated for each individual cultivar, averaged across treatments, and represented as a heat map. Hierarchical clustering was performed using Euclidian distances. Spearman's rank correlation r_s_ (corr) between cooked and raw metabolites color and ellipse eccentricity denote r_s_.

The data was evaluated to understand metabolite correlations between raw and cooked tuber to indicate the potential for analysis of raw tubers to predict cooked tuber phenotypes. Spearman rank correlation was performed on the 85 identified bioactive and nutrient compounds (Table 2 and Figure 6, *r*_*s*_ > |0.205|; *p* < 0.05). Most of the 85 metabolite correlations exhibited a positive relationship (Figure 6, right). Overall, 63 of the 85 compounds (75%) significantly (*p* < 0.05) correlated. The metabolite classes that did not correlate between raw and cooked potato included most amino acids, α-tocopherol 02, and 4-methylumbelliferone (Figure 6). The strongest correlations between raw and cooked potato were for glycoalkaloids, xanthophylls, and chlorogenic acid (Figure 6).

Next, the metabolite data was evaluated within cultivar to understand how metabolites can vary among tubers, within a genotype. Within-cultivar variation was evaluated using coefficient of variation (CV) calculated among *n* = 2 randomly selected tubers per cultivar (Figure 6). The data reveals differences in tuber-to-tuber variation within cultivar for many metabolites. Overall, raw potato tubers had the highest tuber-to-tuber variation, with nutrients having more tuber-to-tuber variation compared to bioactive compounds (Figure 6). Within nutrients, free amino acids were among the classes of compounds with the most tuber-to-tuber variation (Figure 6, bottom). However, several vitamins and bioactives showed little tuber-to-tuber variation, such as chlorogenic acid 02, neoxanthin 02, and glycoalkaloids. Ultimately, metabolites with low CVs and strong correlation values will be ideal targets for future efforts to screen and breed for genotypes with health benefits. Metabolites that meet these criteria include glycoalkaloids, neoxanthin 01, neoxanthin 02, pyridoxal, chlorogenic acid 01 (Figure 6).

### Potato genetics influenced potato mineral content within and among market classes

**Figure 7:**
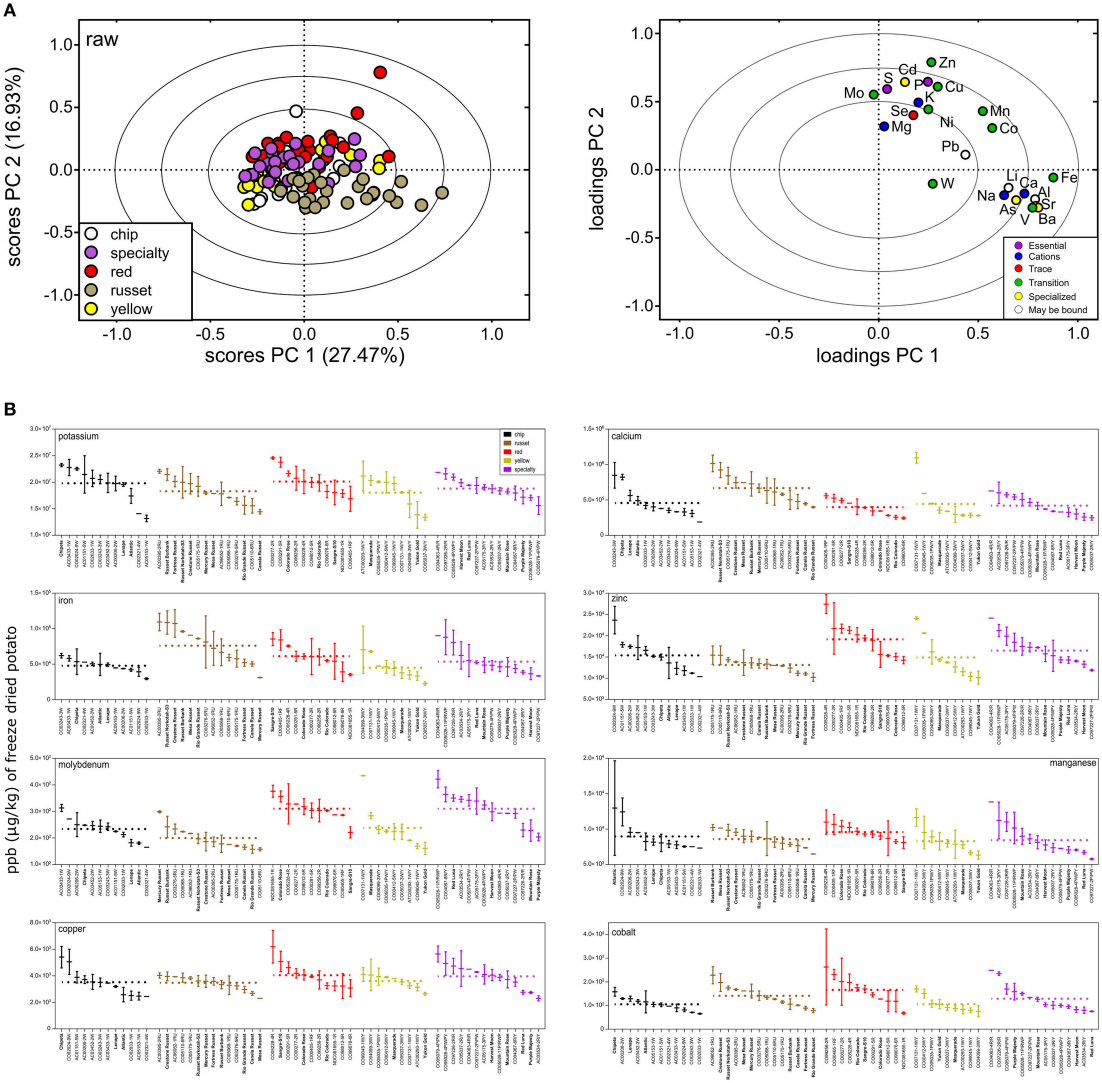
Mineral variation in raw potato tuber. **(A)** PCA was performed on the potato ionome and colored according to potato market class (scores plot, left). PC 1 and PC 2 explained variation among market class for 23 elements (loadings plot, right). The PCA loadings and scores plot were correlation scaled and ellipses denote 0.5, 0.75, and 1.0 correlation values. **(B)** Box and whisker plots of micro- and macronutrient distribution highlight greater variation within potato market class than among potato market class. Potato genotypes within each market class (colored) are arranged from highest mean ppb (μg/kg) of freeze-dried potato to lowest. Mean fold variation within a market class is calculated as mean ppb (μg/kg) of freeze-dried potato of highest potato cultivar within a potato market class divided by mean ppb (μg/kg) of freeze-dried potato of lowest potato variety within a potato market class. Dashed line represents mean ppb (μg/kg) of freeze-dried potato for a market class. Mean fold variation between potato market classes is calculated as mean ppb (μg/kg) of freeze-dried potato of highest potato market class divided by mean ppb (μg/kg) of freeze-dried potato of lowest potato market class. Potato genotypes in bold denote potato cultivars (released commercial varieties). Al, aluminum; As, arsenic; Ba, barium; Cd, cadmium; Ca, calcium; Co, cobalt; Cu, copper; Fe, iron; Pb, lead; Li, lithium; Mg, magnesium; Mn, manganese; Mo, molybdenum; Ni, nickel; P, phosphorous; K, potassium; Se, selenium; Na, sodium; Sr, strontium; S, sulfur; W, tungsten; V, vanadium; and Zn, zinc.

Ionomics analysis was performed using ICP-MS to evaluate variation of minerals in raw potato. A panel of 26 elements were detected, however B, Be, and Cr were below limit of quantification. Similar trends were observed in minerals as with metabolites (Figure 7), specifically for variation among and within market classes. PCA showed separation among potato market classes (Figure 7A) with the largest separation in PC 1 and PC 2 (explained 44.4% of the dataset variability) (Figure 7A). The PCA showed russets to have a more unique mineral profile compare to all other markets classes. This was attributed to increased concentration of iron, calcium, and vanadium and decreased concentration of potassium, zinc, and molybdenum (Figure 7A, right).

For the total potato population, the mineral content varied between 1.7 mFV (Mg) and 5.8 mFV (Ca) (Table 3). Significant variation in potato mineral content was further supported by analysis of variance (ANOVA) that showed mineral variation among market classes (Table 3). Mean elemental concentration for each potato genotype can be found in Supplementary Table 2. Overall, 17 (74%) of the elements significantly varied among market class (ANOVA, FDR-adjusted *p* < 0.05; Table 3). Specifically, iron, zinc, copper, and calcium significantly varied among market class, while magnesium did not (Table 3, Figure 7B). However, as with metabolites, most of the variation was within market class. Overall, element concentration between market classes exhibited 1–2 mFV; whereas mFV within market class ranged from 1 to 7 (Table 3). For example, among market classes Ca exhibited 2 mFV (391, 414, 442, 464, and 662 mg/kg in freeze-dried potato, red, specialty, yellow, chip, and russet, respectively). On the other hand, within market class, Ca exhibit a 4 mFV within chip genotypes and yellow potatoes, a 3 mFV in russet and specialty potatoes, and a 2 mFV within red potatoes (Table 3, Figure 7B, and Supplementary Table 2). Similar trends are observed with potassium: 1 mFV between potato market classes vs. 2 mFV within chip, russet and yellow market classes (Table 3). Interestingly, russet potatoes exhibited a mFV of 4 (31.2 mg/kg in freeze-dried potato, Mercury Russet, to 1,090 mg/kg in freeze-dried potato, AC00395-2RU) in iron content which may allow for increasing potato iron content (Table 3, Figure 7B, and Supplementary Table 2).

**Table 3:** Ionomic composition of raw potato tubers.

**Class**	**Element**	**Atomic symbol**	**ANOVA** ***p*****-values^a^**						**Mean Fold Variation^b^**					
			**Market class^c^**	**Cultivar^d^**					**Market Class^e^**	**Cultivar^f^**				
				**Chip**	**Red**	**Russet**	**Yellow**	**Specialty**		**Chip**	**Red**	**Russet**	**Yellow**	**Specialty**
Essential	Phosphorous	P	^*^	0.09	0.09	0.53	0.67	0.11	1	2	2	1	1	1
	Sulfur	S	^*^	0.33	0.6	0.48	0.32	0.64	1	6	2	2	2	2
Cations	Calcium	Ca	^*^	^*^	^*^	0.13	^*^	0.05	2	4	2	3	4	3
	Magnesium	Mg	0.75	0.17	0.49	0.42	0.25	0.08	1	2	1	2	2	2
	Potassium	K	^*^	0.08	0.25	0.15	0.18	0.07	1	2	1	2	2	1
	Sodium	Na	^*^	0.09	0.18	^*^	^*^	0.06	1	2	2	2	3	2
Trace	Selenium	Se	0.48	1	0.69	0.57	0.63	0.64	1	1	4	1	3	2
Transition	Cobalt	Co	^*^	0.15	0.73	^*^	0.07	^*^	2	2	4	3	2	3
	Copper	Cu	^*^	0.08	0.27	0.26	0.63	0.11	1	2	2	2	2	2
	Iron	Fe	^*^	0.45	0.49	0.24	0.44	0.28	2	2	2	4	3	3
	Manganese	Mn	0.4	0.74	0.74	0.24	0.43	0.08	1	2	1	2	2	2
	Molybdenum	Mo	^*^	0.05	0.42	^*^	^*^	^*^	2	2	2	2	3	2
	Nickel	Ni	0.5	0.17	0.69	0.24	0.63	0.74	1	4	5	2	3	6
	Tungsten	W	0.63	0.98	0.92	0.75	0.61	0.88	1	1	1	1	1	1
	Vanadium	V	^*^	0.35	0.81	0.61	0.74	0.43	2	2	3	3	2	3
	Zinc	Zn	^*^	0.08	0.2	0.45	^*^	0.06	1	2	2	2	2	2
Specialized	Arsenic	As	^*^	0.74	0.27	0.24	0.31	0.07	1	2	3	2	2	1
	Barium	Ba	^*^	0.1	0.09	^*^	^*^	0.28	2	3	3	2	5	4
	Boron	B	1	1	1	1	1	1	0	0	0	0	0	0
	Cadmium	Cd	^*^	^*^	^*^	0.42	^*^	^*^	2	3	3	2	3	3
	Strontium	Sr	^*^	0.08	0.18	^*^	^*^	0.05	2	3	2	2	4	3
May be bound	Aluminum	Al	^*^	0.45	0.49	0.53	0.61	0.64	2	2	2	4	3	3
	Beryllium	Be	1	1	1	1	1	1	0	0	0	0	0	0
	Chromium	Cr	1	1	1	1	1	1	0	0	0	0	0	0
	Lead	Pb	0.16	0.65	0.49	0.75	0.71	0.88	2	3	4	5	7	2
	Lithium	Li	^*^	0.74	0.74	0.12	0.69	0.08	2	2	4	7	2	4

a *Each p-value was calculated using one-way ANOVA and adjusted by a Benjamini-Hochberg correction*.

b *Within market class mean Fold Variation = (potato genotype with the highest element mean peak area)/(potato genotype with the lowest mean peak area). Among market class mean Fold Variation = (potato market class with highest element mean peak area)/(potato market class with the lowest metabolite mean peak area)*.

c *Statistics among potato market class*.

d *Statistics within each market class*.

e *Among market class mean Fold Variation*.

f *Within market class mean Fold Variation*.

* *= p < 0.05*.

## Discussion

Here, non-targeted metabolomics and ionomics was applied to evaluate chemical diversity and quantity of bioactives and nutrients among a diverse set of potato cultivars and advanced lines (Table 1). A biphasic extraction protocol was utilized to optimize the extraction of a wide range of chemical compounds (hydrophilic, amphiphilic, and lipophilic) for detection using multiple mass spectrometry techniques (UPLC- and GC-MS) (62, 76, 77). These strategies allowed for the detection of 2,656 compounds present in the potato tuber metabolome (Figure 1) over ten-fold the number of compounds as previously reported (16, 116, 117).

The analyses revealed compositional variation in raw and cooked potato tuber (Figure 1), both within and among potato market classes (Figures 3, 5, 7). Significant correlations between raw and cooked potato tuber (Figure 6) support the ability to predict cooked potato metabolite content based on raw tuber profiling. In our study, 85 compounds (Table 2) and 23 minerals (Table 3) were identified as nutrients or bioactives that varied within and among potato market classes, supporting the potential for new breeding targets for health.

The bioactive compounds detected in this study have demonstrated effects on human health (Table 2). Many of the metabolites reduce the incidence of a diverse set of chronic diseases and have shown activity against cancer [4-methylumbelliferon, apigenin, rutin, chaconine, solanine, (23, 27–31, 98, 99)], hypertension [oleamide (35), kukoamine (36), kaempferol 3-O-rutinoside (33), chlorogenic acid (37)], diabetes [trigonelline (39), isoferulic acid (101), calystegine A3 (38), calystegine B2 (38, 40–42)], and obesity [neoxanthin (46, 47)]. For example, the polyamine kukoamine has demonstrative hypotensive activity (Table 2) (36). Additionally, the literature indicates that alkaloids such as calystegine A3 and calystegine B2 can be used as therapy against cancer, diabetes, and to stimulate the immune system (38, 48, 118).

While potato genetics is diverse, this sample set was designed to only include genotypes that would be acceptable for commercial fresh market potato consumption. Furthermore, breeding programs and new potato genotypes are monitored for potato glycoalkaloid content due to their toxic effect on humans at high concentrations (48, 83, 119–122). For example, Lenape was removed from the market due to its toxic effects resulting from high glycoalkaloid content (83), but remained in breeding programs due to its agronomic qualities (progeny with high glycoalkaloid content are discarded) (82).

Potatoes contain a large quantity and diversity of glycoalkaloid compounds (Table 2, Figure 5A). These compounds have demonstrated activity in pest and pathogen resistance, are toxic to humans and animals, and impart a bitter taste (48, 83, 120, 122, 123). Regulations, restrictions, and guidelines imposed on potato glycoalkaloid content have resulted in wild potato species with higher glycoalkaloid content when compared to cultivated potato, which highlights the heritability of plant metabolites (119–121). The low tuber-to-tuber variation in our population within a genotype lends credence to this notion (Figure 6) and provides evidence that metabolite content and concentration is under genetic control. While potato glycoalkaloids have demonstrated toxicity, recent studies reveal health-promoting effects and thus desirability. For example, potato glycoalkaloids have chemopreventive effects showing activity against skin, colon, stomach, and liver cancer (27–31, 123).

Low within-cultivar variation further supports the opportunity to breed for enhanced health properties of potato. Metabolites with low CV are ideal targets to screen and breed for health (Figure 6). For example, vitamins such as pantothenic acid, pyridoxal, pyridoxine, and α-tocopherol exhibited low CV (Figure 6). In fact, recent research has determined the ability to breed for potato cultivars with improved vitamin content (124). The antioxidant, chlorogenic acid, is also an ideal target due to little within tuber variability and no impact on abundance or variation occurs with cooking (Figures 2B, 5A, 6, Table 2, and Supplementary Table 1). Furthermore, a recent study demonstrated that the biosynthesis of chlorogenic acid in potato is controlled at the transcriptional level (57, 125).

Yellow potatoes demonstrated very different metabolomes as compared to the other potato market classes (reds, russets, chips, and specialties; Figure 3) with the carotenoid content (lutein and neoxanthin) contributing significantly to this distinction (Figure 3). Potato carotenoid content varied among market class (14 mFV), which supports data from previous studies (59). Yellow potatoes also had reduced lipid content but increased levels of linolenic acid (Figures 3, 5B). This may indicate a metabolic relationship between primary and secondary lipid metabolism during tuber development (e.g., fatty acids and higher-level terpenes).

Potato tuber mineral content also varied (Table 3, Figure 7) within genotype and market class. Importantly, key minerals essential for human development and nutrition (e.g., Fe, Zn, Co, and Ca) significantly varied within potato market class (Table 3 and Figure 7B). For example, iron showed a 3 mFV within potato market classes, which supports data from previous studies (126). The combination of low phytic acid and high ascorbic acid in potato tubers increases iron bioavailability allowing for even small increases in potato tuber iron content to help overcome human iron deficiency (126). Studies focusing on increasing iron content in potato tubers revealed moderate heritability estimates (127). Interestingly, there were strong genetic correlations between multiple micronutrient concentrations (e.g., Fe and Zn) indicating that efforts to breed for Fe can also result in increased levels of the essential mineral Zn (127).

The data presented here demonstrates the breadth of nutrients and bioactive compounds in potato tuber. Cooking had a major influence on phytochemical composition, however many vitamins and bioactive compounds were unaffected or had strong correlations between raw and cooked tuber. These specific vitamins and bioactive compounds are ideal targets for improving the health properties of potato tubers. Taken together, the results of this study support the potential to breed for a healthier potato which could have significant implications in the fight against disease and malnutrition worldwide. Future work is warranted to determine the genetic and environmental factors that mediate chemical diversity in potato.

## Author contributions

JC, DH, CB, JP, and AH wrote the manuscript and had primary responsibility for the final content. DH, JP, and AH conceived the study. JC, CB, and AH collected and analyzed the metabolomics and ionomics data. All authors have read an approved the final manuscript.

### Conflict of interest statement

The authors declare that the research was conducted in the absence of any commercial or financial relationships that could be construed as a potential conflict of interest.

## Supplementary material

Supplementary Figure 1 UPLC-MS chromatogram of the biphasic extraction method. **(A)** Aqueous fraction, **(B)** organic fraction, and **(C)** combined (aqueous and organic) fraction. Arrows denote example differences among chromatograms.

Supplementary Table 1 ANOVA analysis of bioactive and nutrient compounds identified in cooked and raw potato.

Supplementary Table 2 Mean elemental concentration of potato tubers.

## Abbreviations

MeOH: methanol
MTBE: methyl tert-butyl ether
CV: coefficient of variation
UPLC-MS: ultra-performance liquid chromatography-mass spectrometry
GC-MS: gas chromatography-mass spectrometry
ICP-MS: inductively coupled plasma-mass spectrometry
USDA: United States Department of Agriculture
PCA: principal component analysis
PC: principal component
mFV: mean fold variation
ANOVA: analysis of variance.
