# Supplementary figures and images for: Metabolomics and Ionomics of Potato Tuber Reveals an Influence of Cultivar and Market Class on Human Nutrients and Bioactive Compounds
Source: Front Nutr. 2018 May 23;5:36. doi: 10.3389/fnut.2018.00036 (PMC5974217; doi:10.3389/fnut.2018.00036)

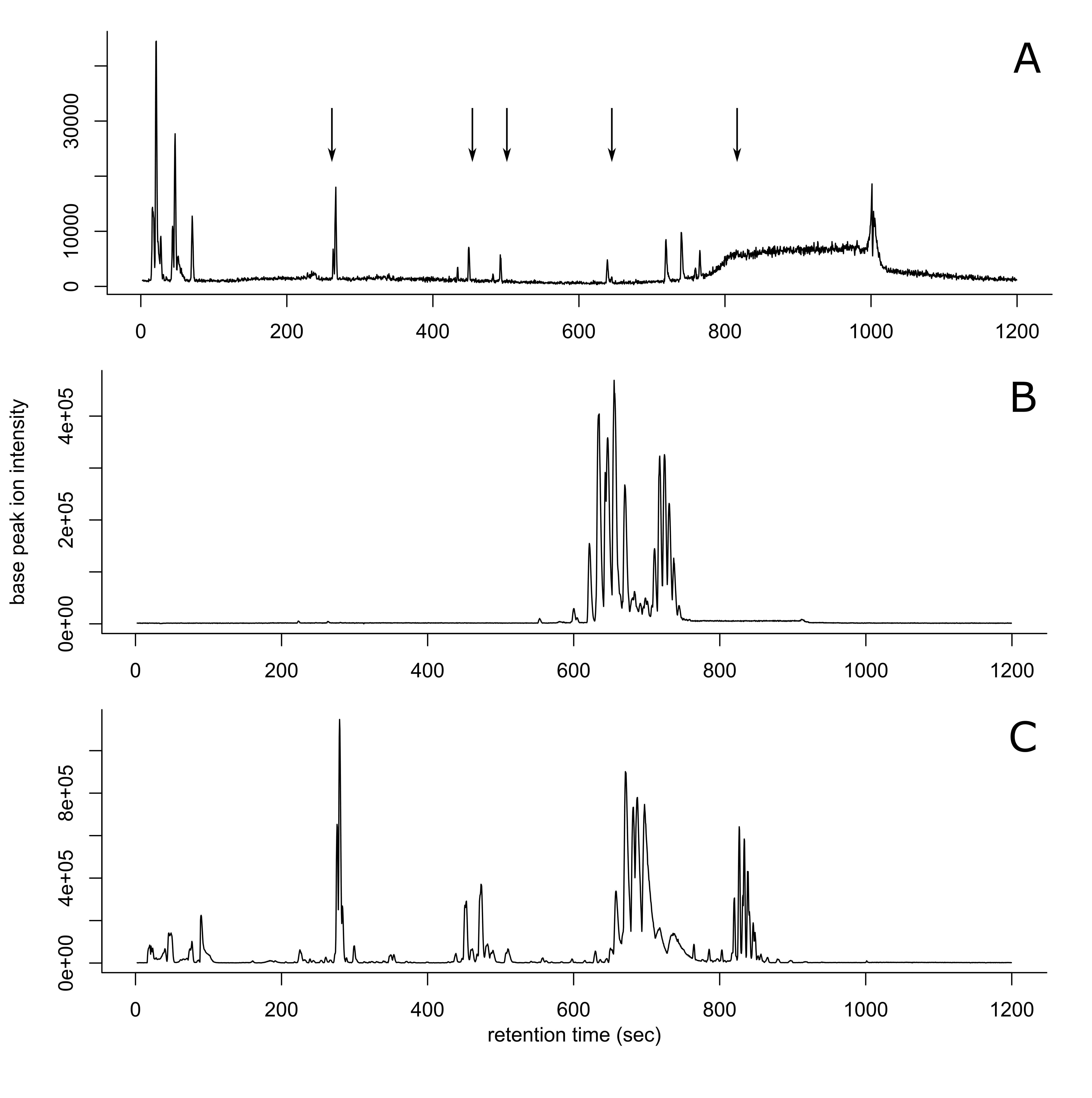

Supplement: Supplementary Figure 1 — UPLC-MS chromatogram of the biphasic extraction method. (A) Aqueous fraction, (B) organic fraction, and (C) combined (aqueous and organic) fraction. Arrows denote example differences among chromatograms. [file Image_1.TIF]
